# The Potential Cell Population Doubling Time in Neuroblastoma and Nephroblastoma

**DOI:** 10.1038/bjc.1971.84

**Published:** 1971-12

**Authors:** W. Aherne, Pamela Buck

## Abstract

Estimates are presented of the median potential cell population doubling time in five neuroblastomas and six nephroblastomas. The median time in the neuroblastomas was 4·2 days (101 hours) and in the nephroblastomas was 7·8 days (188 hours). Estimates of the duration of metaphase in the two kinds of tumour are also offered, based on the natural mitotic index in six neuroblastomas and five nephroblastomas. The findings are set in the context of a brief review of other studies on the cell population kinetics of human tumours. A more detailed but frankly speculative analysis is made of one nephroblastoma which suggests that cells are produced at the rate of *at least* 5 per 1000 tumour cells per hour, of which about 3 are lost.


### THE POTENTIAL CELL POPULATION DOUBLING TIME IN NEUROBLASTOMA AND NEPHROBLASTOMA

W. AHERNE AND PAMELA BUCK

*From the Department of Pathology, Royal Victoria Infirmary, Newcastle upon Tyne NE1 4LP*

Received for publication August 20, 1971

**SUMMARY.—Estimates are presented of the median potential cell population doubling time in five neuroblastomas and six nephroblastomas. The median time in the neuroblastomas was 4·2 days (101 hours) and in the nephroblastomas was 7·8 days (188 hours). Estimates of the duration of metaphase in the two kinds of tumour are also offered, based on the natural mitotic index in six neuroblastomas and five nephroblastomas. The findings are set in the context of a brief review of other studies on the cell population kinetics of human tumours. A more detailed but frankly speculative analysis is made of one nephroblastoma which suggests that cells are produced at the rate of *at least* 5 per 1000 tumour cells per hour, of which about 3 are lost.**

THE study of malignant tumour cell kinetics in man is ethically limited, and it is not surprising that few hard facts have so far been established. Some conclusions, more or less tentative, have been reached, in particular about the rate of cell production, in a few solid tumours and some leukaemias. We do not intend to review these comprehensively in this short paper. Our concern here is to report some observations on the potential cell population doubling time and the rate of cell production in two kinds of embryonal tumours, the neuroblastoma and the nephroblastoma. We shall cite a few typical studies on other solid human neoplasms to indicate prevailing trends.

Refsum and Berdal (1968) measured the average rate of cell proliferation in 61 patients with mainly squamous epithelial neoplasms by using Colcemid to arrest mitosis in metaphase. The rate was such as to indicate a potential cell population doubling time of 2·6 days; the range was 1·0 to 10·8 days. The relationship of this value to the median overall volume doubling time of about 60 days observed in a separate series of cases suggested to them that the loss of cells from the tumour population must have amounted to about 95% of the cells produced. Meyer and Donaldson (1969), also using a stathmokinetic method but with vinblastine as the metaphase blocker, studied four inoperable squamous cell carcinomas of the oral cavity. Biopsies were taken before, during and after intra-arterial or intravenous infusions of vinblastine. Surprisingly, these authors included anaphases with prophases and metaphases in their counts, and assumed that the accumulation of mitotic figures is a linear function of time. Their results for what they termed the generation times (in effect, the potential cell population doubling times) of the tumours they studied were 6, 8, 8 and 9 days.

Some workers have preferred methods based on radioactive labelling. Steel (1967) has summarized and evaluated the published work on the thymidine labelling index (LI) determined *in vitro*, mainly on carcinomas, especially those of the breast, colon and stomach. The median potential cell population doubling time, calculated from

$$T_p = \lambda(t_s/LI); \qquad \lambda = 0{\cdot}75,\ t_s = 15 \text{ hours},$$

was 15·6 days, but of the 10 types listed 5 had a potential population doubling time of less than 10 days. $\lambda$ is a correction factor necessary for exponentially growing cell populations due to the fact that the age distribution graph of the cell cycle is not rectangular and the phase of DNA labelling occurs towards the end of the cycle.

Only in exceptional circumstances can radioactive thymidine be used to investigate human cell population kinetics *in vivo*. Frindel, Malaise, Vassort and Tubiana (1968) have discussed these circumstances. They were able to find only five patients for investigation who satisfied their strict conditions. Four of these had epidermal carcinomas, and the fifth an unspecified malignant tumour of the spinal cord. The experimental procedure, one of the earliest methods devised but still one of the best, was to construct curves showing the percentage of labelled mitoses, counted in serial biopsies, plotted against time after a single injection of tritiated thymidine. They found that the duration of the cell cycle lay between 1 and 4 days.

Most of the kinetic studies of solid tumours in man have been made on those which affect adults. Wagner and Kaser (1970), however, have studied one child with a neuroblastoma. Using a method similar to that of Frindel *et al.* (1968) they found that the cell cycle time was about 40 hours.

### MATERIALS AND METHODS

This report is an account of the findings in a consecutive series of 5 children with neuroblastoma and 6 with nephroblastoma (Wilms). We used a stathmokinetic technique, similar to that of Refsum and Berdal (1968), to determine the rate of cell production *in vivo*. Our patients were given 1–3 mg. Colcemid (CIBA) intravenously, the size of the dose depending on the age of the child. Most of them were children in the first 4 years of life; the oldest was 7 years of age. The median age of the 5 children with neuroblastoma was 5·0 years, and the only tumour which was operable weighed 68 g. The 6 children with nephroblastoma were of median age 2·5 years and the median weight of the 5 operable tumours from them was 740 g.; one child aged 2 years had a tumour weighing 1450 g. The biopsy, or small pieces of the excised tumour, were placed in chilled Susa and the interval between injection of Colcemid and fixation was carefully noted. Paraffin-embedded blocks were processed in the usual way, and 3 $\mu$ sections were stained by the PAS technique, which was found to reveal more precise detail than other stains. Using an eyepiece graticule we counted cells in metaphase as a proportion of all cells.

Since the stathmokinetic method we used cannot provide information about the proportion of cells actually in the proliferative cycle, the proportion or index of arrested metaphases, $I_{\text{met}}(a)$, can only be used to find the potential doubling time $T_p$ of the tumour cell population. This we found by the formula of Puck and Steffen (1963), with $t_a$ (hours) as the interval from injection of Colcemid intravenously to immersion of the biopsy, or pieces of tumour, into cold fixative in the operating theatre:

$$T_p = t_a(0\cdot301)/\log [1 + I_{\text{met}}(a)], \qquad t_a = 4 \text{ hours};$$

assuming, as a reasonable approximation, that the population in each instance was growing exponentially.

Refsum and Berdal's " rate of cell proliferation ", or " rate of cell production " (let us call it $k$) is related to the reciprocal of the potential cell population doubling time, $T_p$. If the rate of cell birth is constant then the rate of cell production, on the exponential assumption, is

$$k = \ln 2/T_p = I_{\text{met}}(a)/t_a.$$

Observations on tumours from 5 children with neuroblastomas and 6 with nephroblastomas, untreated by any metaphase blocker, are included partly to indicate the degree of stathmokinesis we achieved and partly to permit a rough estimate of metaphase duration, $t_{\text{met}}$.

### RESULTS

Tables I and II show the results of metaphase counts made on neuroblastomas and nephroblastomas respectively, both native (a), and accumulated under the stathmokinetic effect of Colcemid (b).

TABLE I(a).—*Neuroblastoma. Metaphase Index* ($I_{\text{met}}$) *of Untreated Tumours*

| | Patient's Age | | | |
|---|---|---|---|---|
| Serial No. | Year | Month | Specimen | $I_{\text{met}}$ |
| 63/3925 | — | — | — | 0·0057 |
| 64/3083 | 2 | 0 | B | 0·0040 |
| 67/3281 | 2 | 0 | T(580) | 0·0068 |
| 67/4783 | 3 | 6 | B | 0·0049 |
| 69/1645 | 4 | 5 | B | 0·0070 |

Median metaphase index: $I_{\text{met}}$ = 0·00570. B: biopsy. T: tumour, with weight in brackets (g.).

TABLE I(b).—*Neuroblastoma. Index of Arrested Metaphases in Tumours Treated* in vivo *by Colcemid*

| | Patient's age | | | |
|---|---|---|---|---|
| Serial No. | Year | Month | Specimen | $I_{\text{met}}(a)$ |
| 68/3741 | 1 | 11 | B | 0·0333 |
| 68/5104 | 1 | 8 | T(68) | 0·0163 |
| 68/5910 | 6 | 0 | B | 0·0420 |
| 69/2750 | 5 | 0 | B | 0·0182 |
| 69/7115 | 7 | 0 | B | 0·0279 |

Median index of arrested metaphases: $I_{\text{met}}(a = 4)$ = 0·0279. B: biopsy. T: tumour, with weight in brackets (g.).

Our patients formed reasonably homogeneous groups, with tumours of comparable sizes and similar degrees of differentiation. It therefore appeared legitimate to average the results. This yielded a median potential cell population doubling time of 101 hours (4·2 days) in the group of neuroblastomas, and 188 hours (7·8 days) in the nephroblastomas.

Estimates of the native metaphase index, $I_{met}$, enabled the duration of metaphase, $t_{met}$, to be approximated from the equation.

$$t_{met} = (t_a . I_{met})/I_{met}(a),$$

where $t_a$ is the period in hours from injection of Colcemid intravenously to immersion of tumour tissue in fixative. In the neuroblastoma group the median duration of metaphase was 0·82 hour (49 minutes). In the nephroblastomas it was 2 hours, which appears unduly long; we need further data to confirm or refute this result.

TABLE II(a).—*Nephroblastoma. Metaphase Index* ($I_{met}$) *of Untreated Tumours*

| Serial No. | Patient's age: Year | Month | Specimen | $I_{met}$ |
|---|---|---|---|---|
| 67/6841 | 1 | 0 | T (700) | 0·0064 |
| 68/0014 | 1 | 10 | B | 0·0095 |
| 68/3472 | 5 | 0 | T (310) | 0·0082 |
| 68/4582 | 4 | 0 | T (170) | 0·0088 |
| 68/10066* | 2 | 6 | — | 0·0061 |
| 70/7242 | 1 | 6 | T (880) | 0·0069 |

Median metaphase index: $I_{met}$ = 0·00755. B: biopsy. T: tumour, with weight in brackets (g.).
* Section submitted from another hospital.

TABLE II(b).—*Nephroblastoma. Index of Arrested Metaphases in Tumours Treated* in vivo *by Colcemid*

| Serial No. | Patient's age: Year | Month | Specimen | $I_{met}(a)$ |
|---|---|---|---|---|
| 69/2790 | 1 | 10 | B | 0·0141 |
| 69/5950 | 3 | 0 | T (680) | 0·0196 |
| 69/6070 | 2 | 0 | T (1450) | 0·0296 |
| 70/0104 | 3 | 0 | T (600) | 0·0101 |
| 70/0409 | 7 | 0 | T (740) | 0·0124 |
| 70/0707 | 1 | 1 | T (939) | 0·0158 |

Median index of arrested metaphases: $I_{met}(a = 4)$: 0·0150. B: biopsy. T: tumour, with weight in brackets (g.).

### DISCUSSION

In approaching the *in vivo* study of tumour cell population kinetics in man one is faced with a rather stark choice of methods. Either one chooses a simple and harmless method, which is applicable in most cases but gives only a first approximation to the truth; or one chooses a refined and penetrating technique, capable of dissecting the cell cycle itself, but which is too dangerous or too disturbing to the patient in ordinary circumstances. We have been compelled by the nature of our material to choose the former; specifically, the enumeration of metaphases arrested by a single dose of the spindle-poison Colcemid.

This method has one major fault. The abnormal metaphases are prone to break up, or become quite pyknotic, and so disappear or at least become unrecognizable for what they are. This naturally leads to underestimation of their true proportion, and so to an overestimation of the potential cell population doubling time, $T_p$. As yet there are no data which would enable the life span of a human arrested metaphase to be estimated but we have found a life span of 4·5 hours (SD $\sim$ 1·5 hours) in an experimental tumour of the hamster. If this, or something like it, is true of the human arrested metaphase also we may have lost about one-third of them by 4 hours. In the case of a typical nephroblastoma from our small group this would reduce $T_p$ from 7·8 days to about 5 days, and in a typical neuroblastoma from 4·2 days to about 3 days. We emphasize, however, that this correction, in the absence of data about the life span of arrested human metaphases, is merely suggestive.

When a tumour appears during infancy or early childhood, and is completely resectable, one is presented with two advantages, of which one at least is usually denied to the student of tumours in adults. It is possible to set a usable upper limit to the age of the tumour; and the total mass of tumour tissue can be assessed. By way of illustration let us take a closer (if somewhat speculative) look at, say, case 70/0707, one of our nephroblastomas. These tumours are much more likely to metastasize to the lungs than the neuroblastomas are. We have in fact made serial measurements radiographically of the growth of the pulmonary metastases

TABLE III.—*Data, Observed and Derived, from a Nephroblastoma Presenting in Late Infancy, Treated by i.v. Colcemid Before Operation*

| | |
|---|---|
| Serial No. . . . . . . . | 70/0707 |
| Age (months) . . . . . . | 13 |
| Maximum age of tumour (days) . . . | 640 |
| Mass of viable tumour tissue (g.) . . . | 470 |
| Approximate mass of single tumour cell (g.) . | $3 \times 10^{-9}$ |
| Metaphase index . . . . . . | 0·0158 |
| Calculated $T_p$ (days) . . . . . | 7·4 |
| Observed $T_D$ (days)* . . . . . | 20 |

* This is a median value from a separate series of 5 cases with measurable pulmonary secondary deposits. The $T_D$ = 20 days is assumed to apply to both 70/0707 and 70/7242.

in 5 nephroblastomas (Aherne and Waddy, unpublished). The median value of the overall volume doubling time, $T_D$, was 20 days. If it is reasonable to put together observations made on different cases of the same embryonal tumour, as we believe it is, then the data shown in Table III may be manipulated to estimate parameters which lie beyond simple stathmokinetic studies.

The data in Table III cannot but be rough estimates. The probable loss of arrested metaphases has not been corrected, the overall volume doubling time is based on secondary deposits, and there are sampling errors in the derived quantities. The mass of the tumour includes connective tissues and blood as well as a large quantity of necrotic material. A Chalkley point count of multiple slices through the tumour in question showed that, on average, about 50% of the tumour mass consists of necrotic material. The mass quoted in Table III is that of viable tumour and its connective tissues. The mass of the whole tumour we have already seen in Table II(b).

We cannot know exactly when the tumour originated or whether it did so in one cell or in a field of cells. The least arbitrary possibilities are that it originated in one cell at the earliest possible time in embryogenesis, namely at about 34 days from conception, in early metanephric tissue. Combining $T_p$, the potential population doubling time, and $T_D$, the overall volume doubling time, gives some idea of the separate rates of cell production ($\beta$) and loss ($\delta$) since, according to the measure of cell loss devised by Steel (1968)

$$\begin{aligned} \phi &= 1 - (T_p/T_D), && \phi \text{ being the `` cell loss factor ''};\\ &= 1 - (7{\cdot}4/20), && \text{from Table III};\\ &= 0{\cdot}63. \end{aligned}$$

But $\phi$ can also be expressed as the ratio of cell loss rate ($\delta$) to cell production rate ($\beta$).

Therefore

$$\delta = 0{\cdot}63\beta.$$

We can now construct a simple approximate model of the growth equation for this nephroblastoma. The model is an exponential one, greatly inferior to, say, a Gompertz or logistic model, but it is the only one which the available data permit. It relates tumour mass, $M_t$ (grams) to time, $t_t$ (days). Tumour volume could, of course, be similarly treated, after the necessary slight correction. The equation states that

$$M_t = M_0 \exp [(\beta - \delta)t]; \qquad \delta = 0{\cdot}63\,\beta, \quad t = 640 \text{ days},$$

and $M_0$ is the cell mass from which the tumour originated. Inserting the appropriate values for case 70/0707 we find

$$\beta = 0{\cdot}004656 \sim 0{\cdot}005, \qquad \delta = 0{\cdot}002933 \sim 0{\cdot}003.$$

This means that *at least* 5 new cells are formed every hour for every 1000 cells in the tumour population. Of these, *at least* 3 cells are lost from the proliferating pool, by necrosis, differentiation or emigration. Unfortunately, the data do not permit an estimation of the size of the proliferating pool, without which it is hardly justifiable to probe any further towards the parameters of the cell cycle itself.

Our thanks are due to Professor A. G. Heppleston, who first stimulated the senior author's interest in this sphere, to Dr. Gordon Steel of the Biophysics Department in the Chester-Beatty Institute for Cancer Research for his interest and constructive criticism, to my colleague Mr. Richard Camplejohn, and to the surgeons who provided the biopsies and excision specimens. The senior author's work has been supported by grants from the Tyneside Leukaemia Fund and Tenovus.

### REFERENCES

FRINDEL, E., MALAISE, E., VASSORT, F. AND TUBIANA, M.—(1968) *Bull. Ass. fr. Étude Cancer*, **55**, 51.
MEYER, J. S. AND DONALDSON, R. C.—(1969) *Archs Path.*, **87**, 479.
PUCK, T. T. AND STEFFEN, J.—(1963) *Biophys. J.*, **3**, 379.
REFSUM, S. B. AND BERDAL, P.—(1968) *Tidsskr. norske Laegeforen.*, **12b**, 1224.
STEEL, G. G.—(1967) *Eur. J. Cancer*, **3**, 381.—(1968) *Cell Tissue Kinetics*, **1**, 193.
WAGNER, H. P. AND KASER, H.—(1970) *Eur. J. Cancer*, **6**, 369.


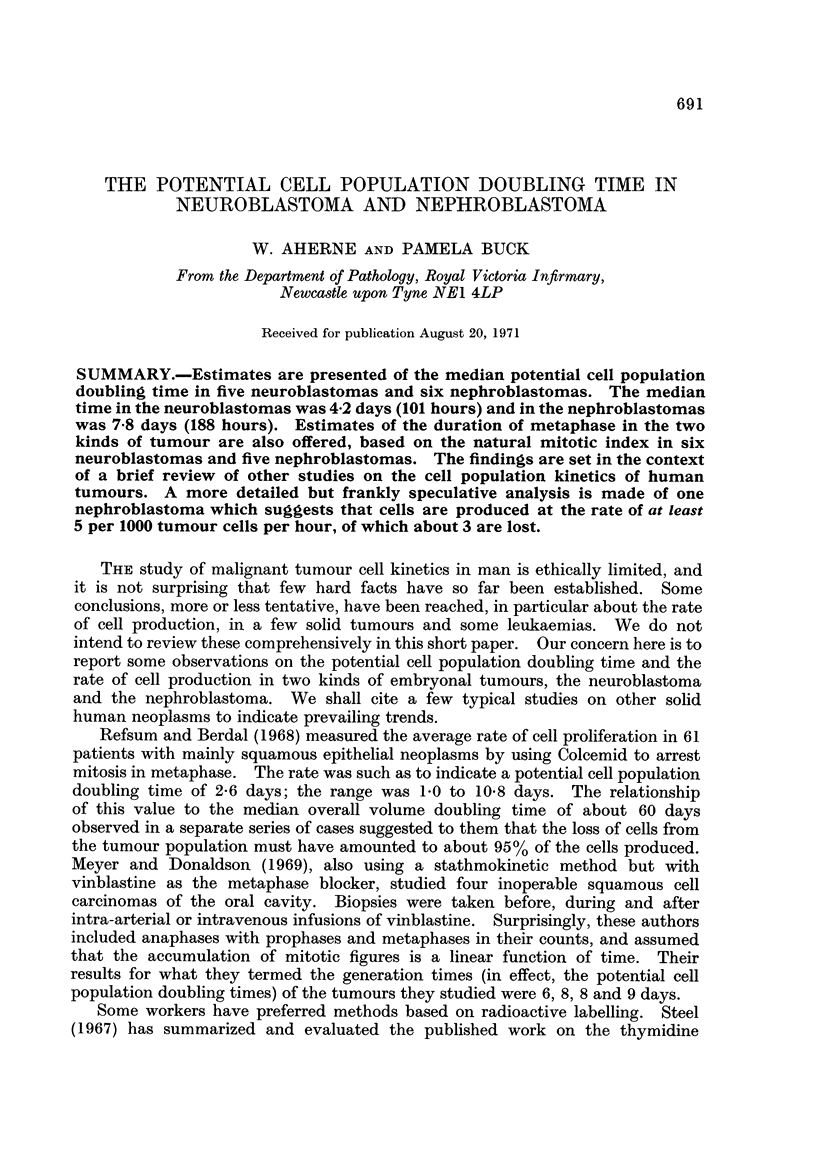

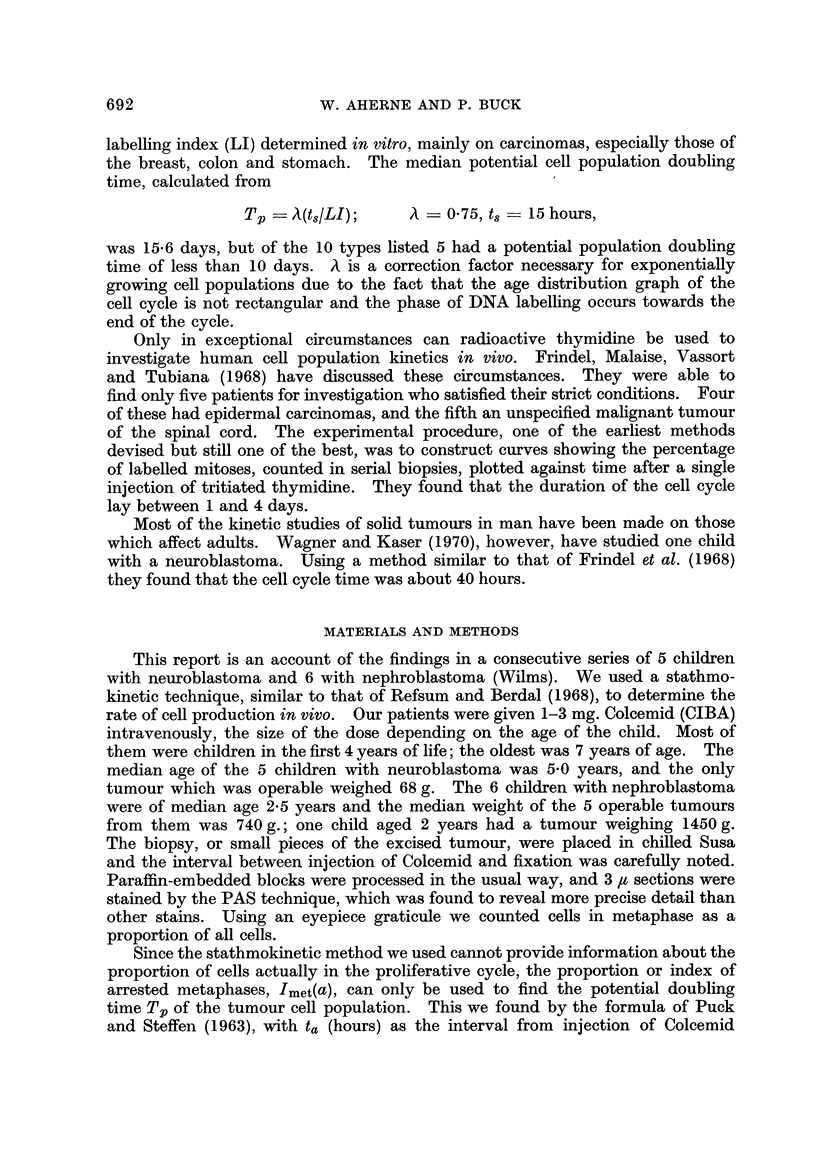

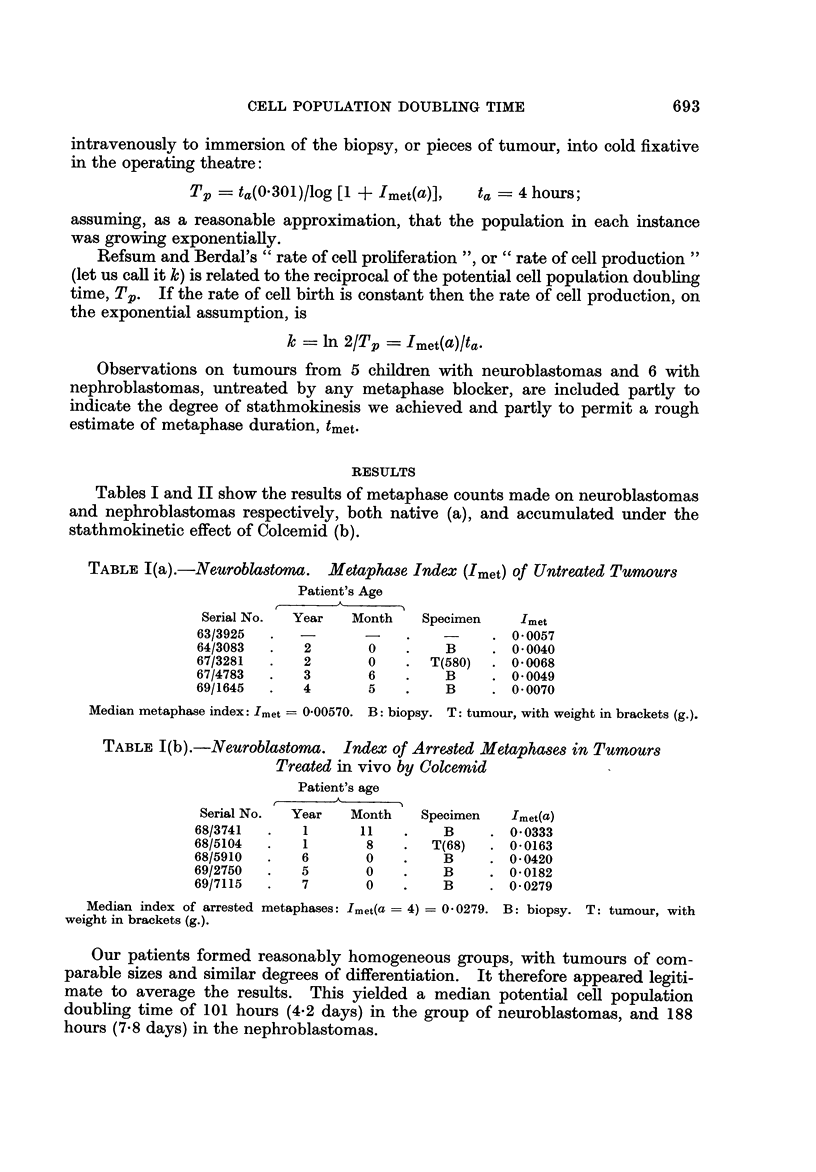

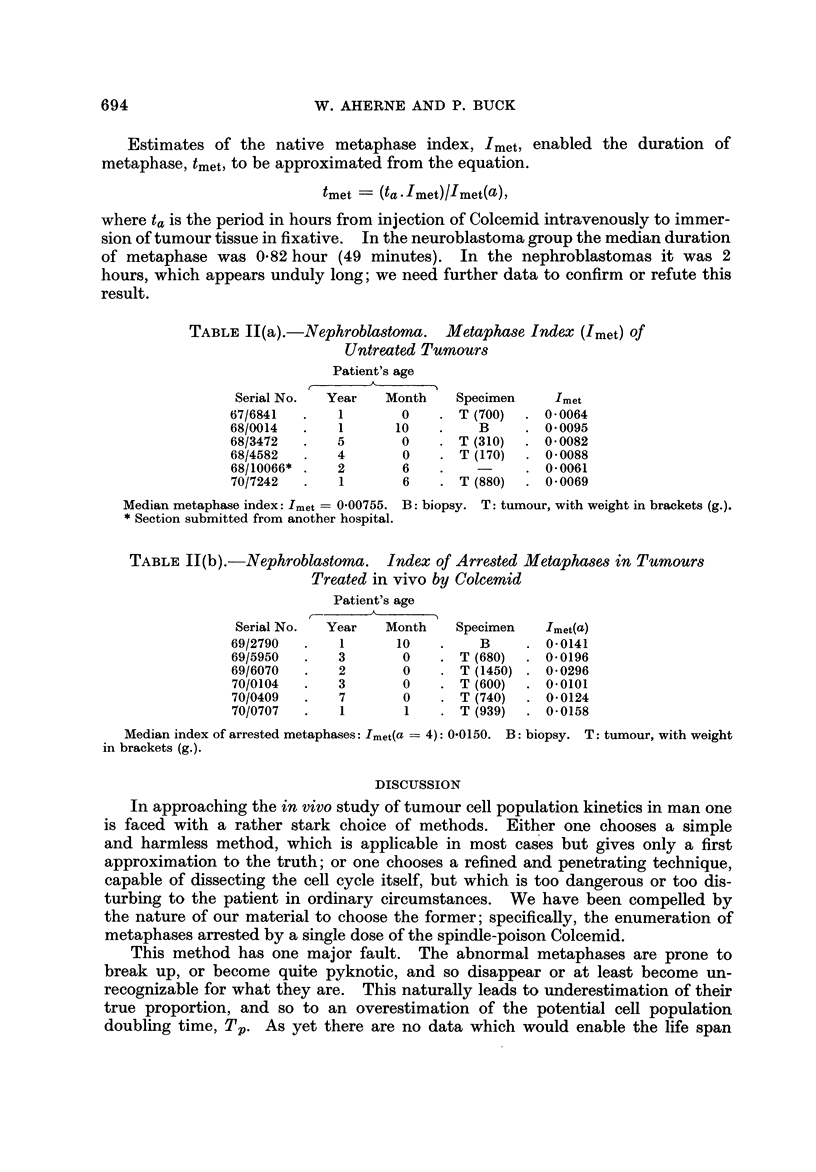

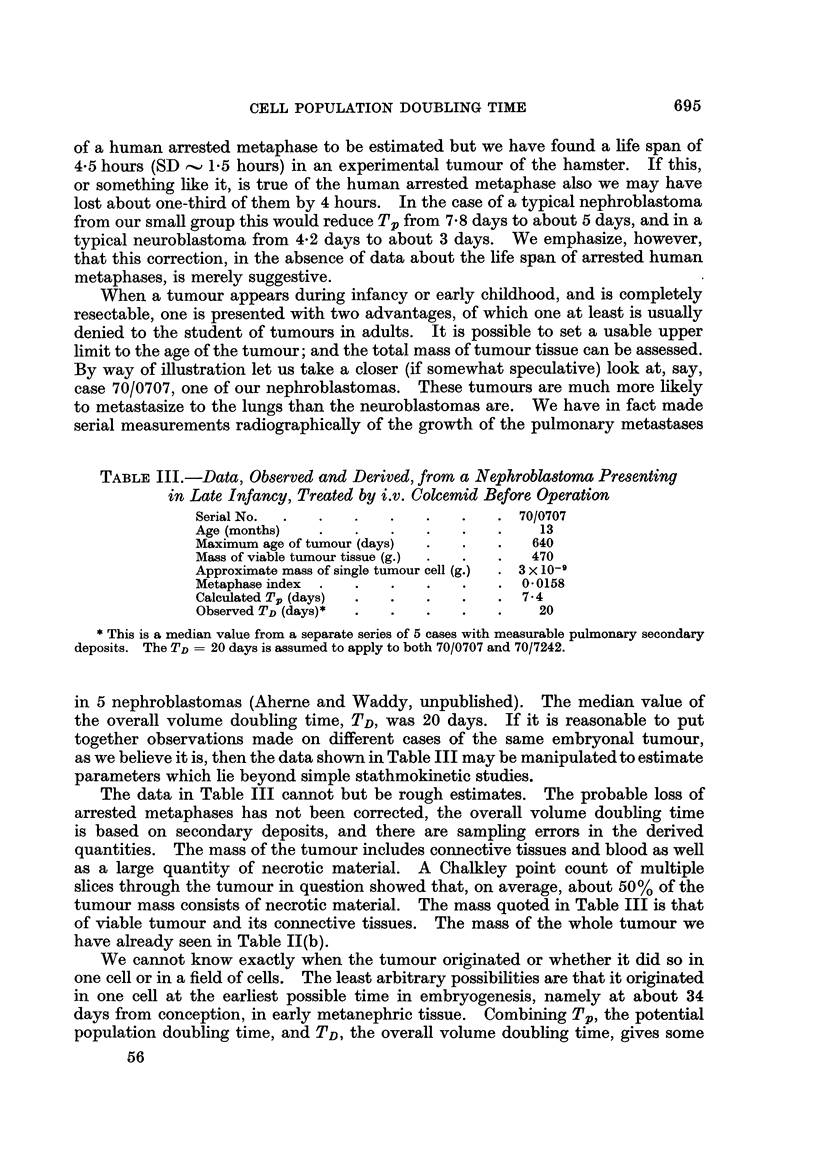

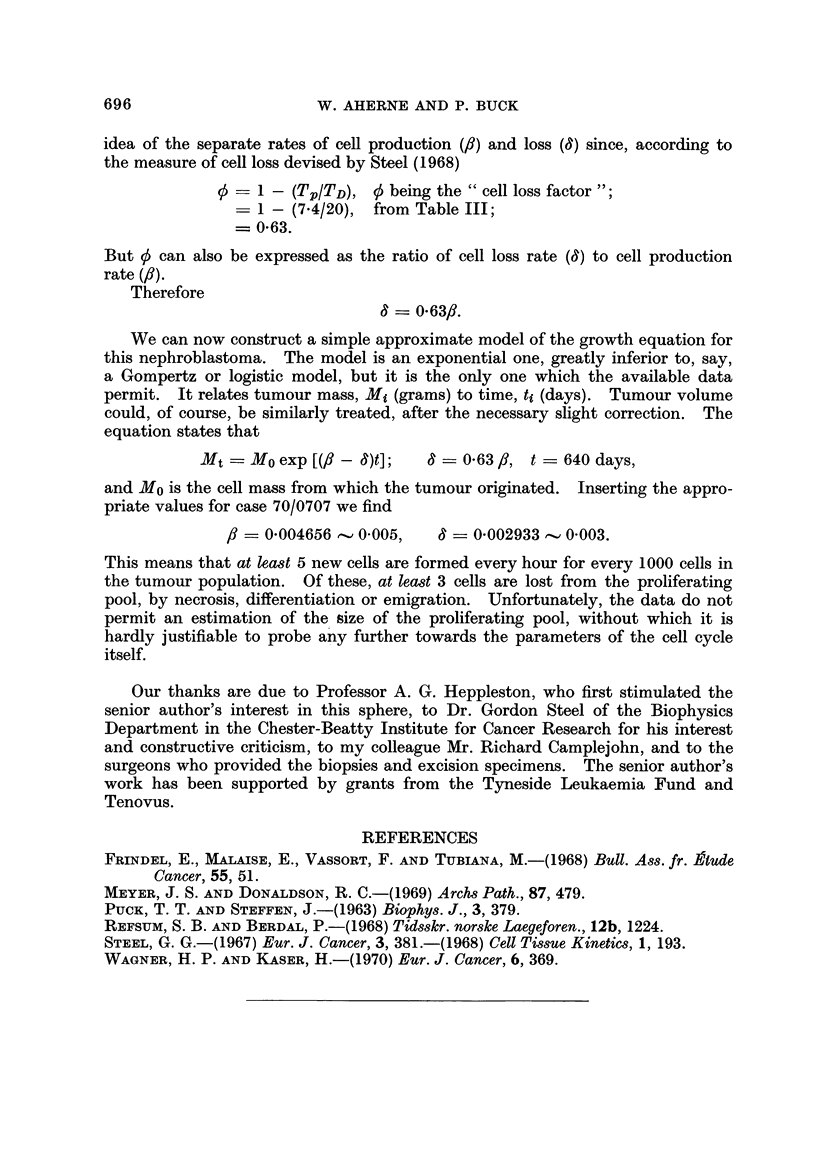


## References

[OCR_00366] Meyer J. S., Donaldson R. C. (1969). Growth kinetics of squamous cell carcinoma in man. A study of four squamous cell carcinomas using stathmokinetic effect of vinblastine in vivo.. Arch Pathol.

[OCR_00367] PUCK T. T., STEFFEN J. (1963). LIFE CYCLE ANALYSIS OF MAMMALIAN CELLS. I. A METHOD FOR LOCALIZING METABOLIC EVENTS WITHIN THE LIFE CYCLE, AND ITS APPLICATION TO THE ACTION OF COLCEMIDE AND SUBLETHAL DOSES OF X-IRRADIATION.. Biophys J.

